# Quality of Life and Associated Factors of International Students in South Korea: A Cross-Sectional Study Using the WHOQOL-BREF Instrument

**DOI:** 10.3390/healthcare10071262

**Published:** 2022-07-06

**Authors:** Chiara Achangwa, Tae-Jun Lee, Junghee Park, Moo-Sik Lee

**Affiliations:** 1 Department of Public Health and Welfare, The Graduate School, Konyang University 709 Ho, Myeongkok Medical Building 158, Gwanjeodong-ro, Seo-gu, Daejeon 35365, Korea; ciaraacha@gmail.com (C.A.); leetj1988@naver.com (T.-J.L.); 2 Myunggok Medical Research Institute, Konyang University College of Medicine, Daejeon 35365, Korea; jhpug@konyang.ac.kr; 3 Department of Emergency Medical Services, The Graduate School, Konyang University 515 Ho, Myeongkok Medical Building 158, Gwanjeodong-ro, Seo-gu, Daejeon 35365, Korea; 4 Department of Preventive Medicine, College of Medicine, Konyang University 705 Ho, Myeongkok Medical Building 158, Gwanjeodong-ro, Seo-gu, Daejeon 35365, Korea

**Keywords:** quality of life, WHOQOL-BREF, associated factors, international students, South Korea

## Abstract

The quality of life (QoL) of an individual is affected in a complex way by the person’s physical health, psychological state, social relationships, and their relationship to their environment. We assessed the QoL of international university students using the World Health Organization Quality of Life (WHOQOL-BREF) tool and explored the QoL-associated factors. We conducted a cross-sectional study between January and March 2021. The WHOQOL-BREF was summarized as a four-domain construct following the WHO guidelines and QoL scores for each domain were compared through a *t*-test and chi-squared test. To determine the factors associated with international students’ QoL we used multiple linear regression models, with each of the domains serving as the outcome variable. In total, 261 students participated, with 52.5% being males. We reported predicted means (PM) along with 95% confidence intervals (CI). Cronbach’s alpha for the overall WHOQOL-BREF tool was 0.88. Students’ self-reported QoL mean score was 3.67 ± 0.71 and the mean score of their overall satisfaction with health was 3.61 ± 0.83. The social relationships domain had the highest mean score at 56.88 ± 19.55 and was significantly associated with age (>25 years: PM: 59.7; 95% CI: 56.2–63.2, *p* = 0.033) and region of origin (Asia: PM: 54.4; 95% CI: 42.5–64.8, *p* = 0.027). Students above 25 years had significantly higher scores in all domains (*p* < 0.05). Our results showed that the social relationships and psychological health domains have more positive impact on international students’ QoL compared to the physical and environmental health domains. To cope with factors influencing international students’ QoL, universities should develop and provide efficient support systems for foreign students in South Korea.

## 1. Introduction

In recent decades, due to globalization, international student mobility from country to country has reportedly increased [1]. Recent statistics show that due to the availability of many scholarships, research funding as well as a conducive study environment, the number of international students hosted by South Korea is growing remarkably, from 83,842 in 2010 to 160,165 in 2019 [2]. These students come from different continents with a great variation in cultures, and languages different from South Korea’s [3]. For international students, while studying in Korea provides the opportunity to obtain an academic degree with rich international experience, it is usually accompanied by many challenges such as homesickness, financial difficulties, and other practical issues associated with a change in the environment, particularly if the international student’s national language is not English, and the culture is very different from that of Korea [4]. The language barrier is one of the major problems for international students in South Korea. Due to their often-limited vocabulary, they usually have difficulties in fully articulating their knowledge leading to difficulties in carrying out academic tasks [5]. The transition to universities in foreign environments can be a stressful experience for students, often requiring substantial psychological adaptation and additional stressors from the process of acculturation [6]. International students are under pressure to succeed, and to live up to the expectations of their families back home. When failure occurs, international students may feel ashamed and isolate themselves from their family and friends, resulting in depression and related psychological problems, with direct negative effects on their overall health and QoL [7]. Even though international students are considered to be a vulnerable group, various sociodemographic factors that affect their QoL remain unclear.

The World Health Organization defines the quality of life as an “individual’s perception of their position in life in the context of the culture, the value systems in which they live and in relation to their goals, standards, expectations, and concerns” [8]. This definition is characterized by individual, and multi-dimensional aspects of the perception of their health and well-being. A widely used generic measure of QoL is the WHOQOL-BREF questionnaire, which is a short version of the WHOQOL-100 instrument [9]. It consists of a total of 26 items, one general QoL, and one health item, as well as 24 specific items with one item from each of the 24 facets of the WHOQOL-100 instrument. To parallel the structure of the WHOQOL-100 and for the purpose of scoring the WHOQOL-BREF, items are combined to form four domain scores, which include physical (dependence medication and treatment, energy and fatigue, sleep and rest, activities of daily living, work capacity, pain and discomfort), psychological (thinking, learning, self-esteem, body image and appearance, negative and positive feelings, spirituality and personal beliefs, memory and concentration), social relationships (social support, personal relationships and sex life), and environment (home environment, financial resources, physical environment pollution, climate and traffic, health and social care, opportunities to acquire new information and skills, leisure, transportation, safety and protection) domains [10]. 

Globally, many studies using different target population samples have shown satisfactory psychometric properties of the WHOQOL-BREF questionnaire [11,12,13,14,15]. A large cross-cultural study carried out in 14 developed and developing countries demonstrated good internal consistency for all domain scores [16]. In South Korea, the assessment of the WHOQOL-BREF in some studies confirmed good internal consistency (Cronbach’s alpha coefficient > 0.7) [9,10]. Since the concept of QoL was accepted, the QoL of many different groups have been evaluated. Although some studies have evaluated the QoL of different groups of students using the WHOQOL-BREF [17,18], we found no study assessing the QoL of international students as a whole in South Korea.

Empirical literature shows that many demographic factors are associated with students’ QoL, including age, gender, and level of study [19,20,21]. According to a study by Lee et al. studying in a new environment coupled with the demanding nature of the curriculum results in mood swings, behavior change, reduced capacity to concentrate, and suicidal ideation [22]. In addition, other factors are reported to be associated with international students QoL [23]. These include study load, number of assignments, deadlines and sponsorship status. These stressors without an appropriate evidence-based solution may contribute to poor QoL as well as compromise the academic performance of international students in South Korea [24]. 

Evaluating the QoL across different youth groups continues to increase in importance for both researchers, and decision makers for policy design, strategy and direction of health programs [25]. Assessing the QoL and its associated factors for international students in South Korea will provide information regarding their present health conditions, perspectives on health and associated factors. This will be beneficial for inclusive health promotion activities for all students in universities in South Korea.

The goal of this study was to assess the QoL of international students in different universities in South Korea using the WHOQOL-BREF tool. In addition, we carried out an exploratory analysis to assess the factors associated with the QoL of international students in South Korea. 

## 2. Material and Methods

### 2.1. Setting and Study Design

We conducted a cross-sectional online study. For student convenience reasons, during the second semester vacation between January and March 2021, male and female international students studying in Universities in South Korea participated in this study. Our inclusion criteria were as follows: students currently studying at any university in South Korea at any level (undergraduate or postgraduate), have spent at least 6 months studying in South Korea, and are able to understand English.

### 2.2. Participants and Sample Size

We used snowball sampling methodology [26] to send the survey questionnaire to potential participants. This method entailed sending the survey link with the inclusion criteria via E-mail, WhatsApp, Kakao Talk, and other social media platforms to international students in universities in South Korea. We then requested recipients of the study questionnaire to disseminate it further within their networks of international students. Due to the various techniques used to disseminate the survey link, we were unable to precisely quantify recipients and track response rates as per American Association for Public Opinion Research (AAPOR) reporting guideline [27]. We calculated our sample size using the R statistical software. To calculate our sample size, we used the international student population of 160,000 [2], a standard deviation of 4.8 [23], and a precision of 0.6. This yielded a minimum sample size of 246. 

### 2.3. Survey Tools

#### 2.3.1. Sociodemographic Variables

This study had questions on the age, gender, level of study, monthly income in Korea, study load, sponsorship, region, and marital status.

#### 2.3.2. The WHOQOL-BREF Questionnaire

WHOQOL-BREF questionnaire, a brief version of the WHOQOL-100 was used to assess the QoL [28]. WHOQOL-BREF consists of 26 items classified into four domains: physical health, psychological health, social relationships, and environment. Item one and item two separately assessed the overall perception of QoL and satisfaction with health. Each item was rated on a 5-point Likert scale from 1 (very poor/very dissatisfied/never/none) to 5 (very good/very satisfied/always/extremely). Raw domain scores for the WHOQOL were transformed to a 4–20 score which was then normalized into a score from 0 to 100, with higher scores indicating better QoL. Many studies have evaluated the validity and reliability of the WHOQOL-BREF instrument and found it suitable for evaluating the QoL [29].

### 2.4. Statistical Analysis

Entries from the online surveys were recorded in google sheets, cleaned, and exported to R version 4.0.3 package for analysis. We conducted univariate analysis for all the variables, and examined all variables visually and statistically for normality of distribution and any measurement errors; however, there were no values outside the expected range for each variable. Our analysis was divided into three parts. Firstly, we tested the internal consistency of our study tool by computing the Cronbach’s alpha coefficient. Cronbach’s alpha values equal to or greater than 0.70 were considered satisfactory. Secondly, descriptive statistics were computed for all variables. Numerical variables were summarized as a mean ± standard deviation (SD), and categorical variables were summarized by frequency and percentage. The WHOQOL-BREF was summarized to a four-domain construct (physical health, psychological health, social relationships, and environment) following the WHO guidelines to calculate the mean domain scores. QoL scores for each domain was compared through *t*-test and chi-squared test. QoL scores were computed for different subgroups (age, gender, level of study, study load, and sponsorship) and the domain scores difference between each subgroup was computed using the chi-square test.

Finally, we used multiple linear regression models, with each of the domains serving as the outcome variable to determine any significant association between sociodemographic variables, and the QoL domains. Because the outcomes were numeric, we reported the predicted means (PM) with 95% confidence intervals (CI). All statistical tests were two-tailed and the level of statistical significance was set at *p*-value < 0.05.

## 3. Results

### 3.1. Study Participant’s Characteristics

A total of 261 students, 52.5% males and 46.7% females from 63 universities responded to the questionnaire. We categorized the ages of the study respondents using the median (25 years) as the cut-off point; most respondents (54.8%) in this study were in the age group ≤ 25 years. Among all the respondents, 56.7% were studying for a master’s degree, 95% were full-time students, and 86.6% were on scholarship. Of all the respondents, 14.6% were married, 59.4% had monthly income of over one million won, and by region of origin, 48% were from Asia, 37% from Africa, 9.2% from Europe and 5% from America. 

With respect to the self-assessment conducted by international students in universities in South Korea, the mean score of their overall self-reported QoL was 3.67 ± 0.71. Generally, 56.3% of international students described their QoL as good, 8.1% as very good, and 30.7% as neither good nor poor, while 4.6% felt their QoL was poor and 0.4% felt it was very poor. On the other hand, the mean score of their self-rated satisfaction with current health was 3.61 ± 0.83. Most were satisfied with their health (51%), 10.7% were very satisfied, 27.6% were neither satisfied nor dissatisfied, 10.3% were dissatisfied and 0.4 reported being very dissatisfied with their health.

### 3.2. Tool’s Reliability

The level of internal consistency for the WHOQOL-BREF tool (all 26 items) measured by Cronbach’s alpha coefficient was 0.88 for this study. For the physical domain it was 0.87, 0.88 for the psychological health domain, 0.89 for social relationship domains, and 0.88 for environmental domain. All the scores were above the set 0.7 score, indicating good internal consistency in all domains.

### 3.3. Overall WHOQOL-BREF Scores

With regard to QoL domains and as shown in Table 1, the social relationships domain had the highest mean score at 56.88 ± 19.55, followed by the psychological health domain at 56.35 ± 18.06, the physical health domain 50.85 ± 18.41 and finally the environmental health domain at 43.67 ± 15.47 (Table 1). We also conducted paired *t*-tests to compare the means of the four domains. The social relationships domain mean was significantly higher than the means of the three other domains (*p* < 0.001). The mean of the psychological domain was significantly higher than the mean for the physical domain (*p* < 0.002). The physical domain was significantly higher than the environment domain (*p* < 0.001).

### 3.4. WHOQOL-BREF Scores by Subgroups 

When comparing international students QoL in terms of sociodemographic factors, we observed significant differences as follows:

Participants in the age group above 25 years of age had significantly higher mean scores in all domains compared to those who were 25 years or less of age. In addition, males had significantly higher domain scores in all domains except in the social relationships domain compared to females. Students at the doctoral level of studies had significantly higher mean domain scores in the psychological domain (*p* = 0.04) and the environmental domain (*p* = 0.003) compared to students in other levels of studies. Students who were married had significantly higher psychological domain scores compared to singles (*p* = 0.04). Full-time students had significantly higher domain scores in the psychological domain (*p* = 0.044) compared to part-time students. Finally, students on scholarship presented with significantly higher mean scores in the psychological domain (*p* = 0.045) and environmental domain (*p* = 0.005) compared to self-sponsored students (Table 2).

### 3.5. Associations between QoL of International Students and Sociodemographic Factors

To determine the factors associated with the QoL among international students, we developed multiple linear regression models, with each of the domains serving as the outcome variable. The results of the multiple regression analysis are presented in Table 3.

## 4. Discussion

Our results show that the WHOQOL-BREF instrument was reliable for assessing the QoL of international students studying in universities in South Korea. This was similar to many studies which have reported reliability with WHOQOL-BREF for QoL assessment [30,31,32]. In our study, the social relationships and psychological health domains had higher mean domain scores. These results suggest that the social relationships and psychological health domains may have a more positive impact on international students’ QoL than the physical and environmental health domains. These results are similar to the results of a study by Vo et al., who also reported higher scores in the social relationships and psychological health domains among students in Ho Chi Minh in Vietnam [33]. Contrary to our findings, some studies reported rather higher scores in the physical domain [34,35]. Several factors could explain these observed differences. Our study was conducted during the coronavirus disease 2019 (COVID-19) era, with unstable extrinsic environmental factors caused by the pandemic control measures such as social distancing and school shutdown which could affect the physical domain [36]. In addition, this difference could be due to the multicultural nature of our study sample and also because of the economically stable environment and well-balanced society that provides support to international students’ social as well as psychological wellbeing in South Korea.

Exploring the QoL scores among international students, we found that being older in age significantly affected all QoL domains. Older students may easily adapt to studying in a new environment compared to younger students. Our findings are contrary to the study by Chai et al., who found domain scores not significantly affected by age [37]. Males presented with significantly higher physical, psychological, and environmental health domain scores compared to females. Our results could suggest that male students moving to South Korea may feel more prepared than female students to deal with various challenges related to moving to a new environment. Students at the doctoral level of study had significantly higher psychological and environmental health domain scores compared to students at other levels. This is probably because of the high degree of confidence exhibited by most doctoral students due to the increased number of academic and research experiences. Doctoral students can easily interpret or fit into academic and research projects as opposed to undergraduates who have no mastery of various subject matter. However, these results were contrary to some findings which reported lower domain scores for doctoral students as a result of psychological burnout [38,39]. Part-time students had significantly lower psychological domain scores than full-time students. This could be due to the combined stress acquired from studying and working. Hectic routine working schedules involving long hours, difficult tasks and short breaks can result in occupational stress [40]. Part-time students with increased occupational stress are likely to have lower QoL. Universities in South Korea need to pay attention to part-time student’s psychological needs and provide them the required psychological counseling and financial support where need be, in order to boost their psychological health and QoL.

In this study, the sociodemographic characteristics of international students in South Korea were used to predict each of the WHOQOL-BREF domain scores. Factors that were associated with the physical health domain were age, gender, and region of origin, while factors associated with the psychological health domain included age, gender, being single, region of origin, level of study and study load. Factors that influenced the social relationships domain were age and region of origin, and those that affected the environmental domain were age, gender, level of study, region of origin and sponsorship. Many factors are reported to influence students’ QoL, including study load, gender, sponsorship, marital status, and age [41].

The strength of this study are as follows: it provides evidence of the QoL and associated factors among international students in South Korea using an internationally validated instrument—WHOQOL-BREF. Additionally, participants were from 63 universities, which is demographically diverse enough to be representative of all international students in South Korea. However, our study had a few limitations. First, our sample did not consider a minimum participation number of students from each continent, making it difficult to appreciate cultural differences. In addition, this was a cross-sectional study, and hence, it did not account for any change over time, neither could we infer any causal relationships. Additionally, as data for this study were self-reported, some level of bias may be present (recall bias). However, we anticipate that this bias is minimal. This study did not take into consideration university representation, which to some extent may affect the student’s QoL. Using the models, we attempted to control for biases, such as location of student’s institution, and region of origin, but there might be residual confounding. Furthermore, this study could be improved with the inclusion of some variables that were not assessed, such as satisfaction with the course and the career choice, the curriculum, and academic performance, which could have an effect on the QoL of students. This study also did not assess the health of international students prior to their relocation to South Korea. Therefore, it was not possible to know whether the QoL was due to the influence of events occurring prior to relocation. This information could be important while designing health promotion activities as to whether or not to monitor students’ health status after arrival.

## 5. Conclusions

This study assessed the QoL and explored the factors associated with the QoL of international students in South Korea. Beyond ascertaining the validity of the WHOQOL-BREF instrument, the results presented demonstrate the complexity of the relationships between the different domain scores and the various sociodemographic factors.

In this study, most international students in South Korea described their overall QoL and health as good and satisfactory, respectively. However, this finding was contrary to other previous studies that had found a lower QoL in international students as a result of acculturation stress. Older international students have better QoL compared to younger students. The psychological domain scores were found to be significantly lower for part-time and self-sponsored students than for full-time students and those on scholarship. This study provides further insights on the current perceived QoL, as well as on relevant factors affecting students’ QoL. 

The findings indicate that programs that address student welfare in Korea should promote international student-friendly atmospheres which could help to improve the subjective QoL of the students. South Korean public health and academic authorities might consider providing more support for part-time students, self-sponsored students as well as students below 25 years of age. Promoting international students’ QoL in South Korea is not only important for designing health promotion activities in universities in South Korea, but can also result in better student performance and welfare.

Future studies could examine the WHOQOL-BREF domain scores of international students across various majors to determine if student’s perception of QoL varies among different fields of study at the university level. Further studies are also recommended to be carried out on subgroups which showed a lower domain score in order to establish reasons for poor QoL.

## Figures and Tables

**Table 1:** Participants’ overall scores on WHOQOL-BREF domains.

Domains	Number of Items	Mean Score ± SD *	Min	Max	Cronbach Alpha
Physical health	7	50.85 ± 18.41	0.00	100.00	0.87
Psychological health	6	56.35 ± 18.06	0.00	100.00	0.88
Social relationships	3	56.88 ± 19.55	0.00	100.00	0.89
Environmental health	8	43.67 ± 15.47	0.00	100.00	0.88

* A higher score represents better condition (scores range from 0 to 100).

**Table 2:** QoL domain scores according to sociodemographic factors.

Variables	Physical Health		Psychological Health		Social Relationships		Environment	
	Mean ± SD	*p*-Value	Mean ± SD	*p*-Value	Mean ± SD	*p*-Value	Mean ± SD	*p*-Value
Age								
≤25	48.5 ± 17.3	0.023	52.5 ± 17.2	<0.001	54.5 ± 17.8	0.037	41.7 ± 14	0.026
>25	53.7 ± 19.4		61 ± 18.1		59.7 ± 21.2		46.1 ± 16.8	
Gender								
Male	53.6 ± 17.1	0.018	60.6 ± 18.2	<0.001	57.3 ±19.9	0.796	46.3 ± 15.9	0.005
Female	48.2 ± 19.3		51.9 ± 16.7		56.7 ± 19.1		41 ± 14.3	
Marital status								
Married	54.4 ± 16.1	0.159	61.9 ± 17.4	0.04	59.8 ± 20.5	0.341	46.5 ± 15.8	0.242
Single or divorced	50.2 ± 18.7		55.4 ± 18		56.4 ± 19.4		43.2 ± 15.4	
Region of origin								
Africa	55.3 ± 16.9	0.001	63.8 ± 18	<0.001	60.2 ± 21	0.108	46.7 ± 16.2	0.096
Americas	40.2 ± 16.2		50.9 ± 20.5		62.2 ± 18.1		43.2 ± 9.31	
Asia	50 ± 19.4		51.7 ± 16.3		54.4 ± 18.3		42 ± 15.7	
Europe	43.1 ± 14.9		53.2 ± 16.4		53.4 ± 19.2		40.1 ± 12.4	
Level of study								
Bachelor	49.9 ± 18.7	0.685	51.9 ± 17.6	0.04	53.7 ± 19.5	0.245	38 ± 15.2	0.003
Master	50.8 ± 19.2		57.6 ± 18.2		57.4 ± 18.8		45.5 ± 15.4	
Doctoral or higher	52.7 ± 15.6		59.6 ± 16.6		60.1 ± 21.6		46.1 ± 14.4	
Monthly income in Korea								
Less than 1 million won	51.8 ± 18.2	0.475	56.3 ± 19.4	0.988	56.3 ± 20.9	0.68	42.8 ± 14.9	0.43
More than 1 million won	50.2 ± 18.5		56.4 ± 17.2		57.3 ± 18.6		44.3 ± 15.9	
Study load								
Full-time	50.9 ± 18.5	0.856	56.9 ± 17.9	0.044	57.1 ± 19.5	0.5	44 ± 15.5	0.115
Part-time	50 ± 17		45.1 ± 18.8		53.1 ± 19.9		37.5 ± 13.5	
Sponsorship								
Scholarship	50.4 ± 18.7	0.24	57.2 ± 18.2	0.045	57.6 ± 19.7	0.132	44.5 ± 15.8	0.005
Self-sponsor	54 ± 16.3		51 ± 16.2		52.5 ± 18.1		38.1 ± 11.5	

We evaluated statistical significance using *t*-tests for numeric variables and chi-square tests for categorical variables and level of significance was considered at *p* < 0.05, SD: Standard deviation.

**Table 3:** Associations between quality of life of international students and sociodemographic factors.

Predictors	Physical Health Domain, PM(CI) [*p*-Value]	Psychological Health Domain, PM(CI) [*p*-Value]	Social Relationships Domain, PM(CI) [*p*-Value]	Environmental Domain, PM(CI) [*p*-Value]
Age				
≤25	48.5 (45.5–51.5) [Ref]	52.5 (49.7–55.4) [Ref]	54.5 (51.4–57.7) [Ref]	41.7 (39.2–44.2) [Ref]
>25	53.7 (50.4–57) [0.022]	61.0 (57.8–64.1) [<0.001]	59.7 (56.2–63.2) [0.033]	46.1 (43.3–48.8) [0.024]
Gender				
Female	48.2 (45–51.4 [Ref]	51.9 (48.8–55) [Ref]	56.7 (53.2–60.2) [Ref]	41 (38.3–43.7) [Ref]
Male	53.6 (50.6–56.6 [0.017]	60.6 (57.7–63.5) [<0.001]	57.3 (54.1–60.6) [0.797]	46.3 (43.7–48.8) [0.005]
Marital status				
Married	54.4 (48.5–60.2) [Ref]	61.9 (56.2–67.6) [Ref]	59.8 (53.6–66) [Ref]	46.5 (41.5–51.4) [Ref]
Single or divorced	50.2 (47.8–52.7) [0.201]	55.4 (53.1–57.8) [0.041]	56.4 (53.9–59) [0.318]	43.2 (41.2–45.2) [0.23]
Region of Origin				
Africa	55.3 (52.4–59.6) [Ref]	63.8 (55.3–66.9) [Ref]	60.2 (54.5–65.9) [Ref]	46.7 (41.3–58.2 [Ref]
Americas	40.2 (38.1–42.7) [0.005]	50.9 (48.7–53.2) [0.012]	62.2 (55.4–65.9) [0.723]	43.2(39.8–46.7) [0.434]
Asia	50 (40–60) [0.027]	51.7 (48.3–55.4) [<0.001]	54.4(42.5–64.8) [0.027]	42.0 (39.8–44.7) [0.023]
Europe	43.1 (42.1–45.7) [0.003]	53.2(42.5–63.8) [0.007]	53.4(49–58.4) [0.125]	40.1 (38.3–42.7) [0.058]
Level of study				
Bachelor	49.9 (45.4–54.4) [Ref]	51.9 (47.6–56.2) [Ref]	53.7 (49–58.4) [Ref]	38 (34.3–41.6) [Ref]
Doctoral or higher	52.7 (47.3–58.3) [0.441]	59.6 (54.2–64.8) [0.025]	60.1 (54.4–65.9) [0.091]	46.1 (41.3–50.2) [0.006]
Masters	50.8 (47.8–53.7) [0.761]	57.6 (54.7–60.5) [0.031]	57.4 (54.3, 60.6) [0.199]	45.5 (43–47.9) [<0.001]
Monthly income in Korea				
Less than 1 million won	51.8 (48.3–55.3) [Ref]	56.3 (52.9–59.8) [Ref]	56.3 (52.5–60) [Ref]	42.8 (39.8–45.7) [Ref]
More than 1 million won	50.2 (47.8–53.7) [0.477]	56.4 (53.5–59.3) [0.988]	57.3 (54.5–60.8) [0.673]	44.3 (42–46.9) [0.435]
Study load				
Full time	50.9 (48.6–53.2) [Ref]	56.9 (54.7–59.2) [Ref]	57.1 (54.6–59.5) [Ref]	44 (42.1–45.9) [Ref]
Part-time	50 (40–60) [0.865]	45.1 (35.3–54.8) [0.02]	53.1 (42.5–63.8) [0.481]	37.5 (29.1–45.8) [0.137]
Sponsorship				
Scholarship	50.4 (48–52.8) [Ref]	57.2 (54.8–59.5) [Ref]	57.6 (55–60.1) [Ref]	44.5 (42.5–46.5) [Ref]
Self-sponsor	54 (47.9–60.1) [0.283]	51 (45–56.9) [0.059]	52.5 (46–58.9) [0.152]	38.1 (33.1–43.2) [0.023]

Ref = referent, PM = predicted means, CI = confidence interval: multiple linear regression model *p*-value was set at <0.05.

## Data Availability

Data will be made available upon request. Please contact the corresponding author of this article and data will be provided to you within 24 h. The data are not publicly available due to privacy reasons.
